# Is There a Sampling Bias in Research on Work-Related Technostress? A Systematic Review of Occupational Exposure to Technostress and the Role of Socioeconomic Position

**DOI:** 10.3390/ijerph18042071

**Published:** 2021-02-20

**Authors:** Prem Borle, Kathrin Reichel, Susanne Voelter-Mahlknecht

**Affiliations:** 1 Institute of Occupational Medicine, Charité-Universitätsmedizin Berlin, Corporate Member of Freie Universität Berlin, Humboldt-Universität zu Berlin, and Berlin Institute of Health, Augustenburger Platz 1, 13353 Berlin, Germany; Susanne.Voelter-Mahlknecht@charite.de; 2 Independent researcher in occupational health, 10245 Berlin, Germany; kareichel@gmx.de

**Keywords:** socioeconomic status, occupational status, digital divide, precarious labour, platform work, digitalisation, health inequalities, workplace well-being, knowledge workers, sampling bias

## Abstract

Technostress is a widespread model used to study negative effects of using information communication technologies at work. The aim of this review is to assess the role of socioeconomic position (SEP) in research on work-related technostress. We conducted systematic searches in multidisciplinary databases (PubMed, PubMed Central, Web of Science, Scopus, PsycInfo, PsycArticles) in June 2020 and independently screened 321 articles against eligibility criteria (working population, technostress exposure, health or work outcome, quantitative design). Of the 21 studies included in the narrative synthesis, three studies did not collect data on SEP, while 18 studies operationalised SEP as education (eight), job position (five), SEP itself (two) or both education as well as job position (three). Findings regarding differences by SEP are inconclusive, with evidence of high SEP reporting more frequent exposure to overall technostress. In a subsample of 13 studies reporting data on educational attainment, we compared the percentage of university graduates to World Bank national statistics and found that workers with high SEP are overrepresented in 11 of 13 studies. The resulting socioeconomic sampling bias limits the scope of the technostress model to high SEP occupations. The lack of findings regarding differences by SEP in technostress can partly be attributed to limitations in study designs. Studies should aim to reduce the heterogeneity of technostress and SEP measures to improve external validity and generalisability across socioeconomic groups. Future research on technostress would benefit from developing context-sensitive SEP measures and quality appraisal tools that identify socioeconomic sampling biases by comparing data to national statistics.

## 1. Introduction

Historically, the widespread perception that the digitalisation of everyday life and work is constantly increasing to unprecedented levels is not new [1]. Given how hard it is to imagine working without the use of computers, emails and the internet, the wide range of work-related use of information and communication technologies (ICT) can quickly become taken for granted. Yet, political and economic commentators often frame social and technological changes as either a problem or a solution when they summon the buzzword digitalisation. Such debates regarding today’s workplaces then try to evaluate digitalisation in very broad strokes, mostly deeming it either good or bad [2].

The research term technostress has gained increasing popularity in various disciplines such as information systems, psychology and public health to name adverse effects of digitalisation in the workplace and beyond. In what follows, we understand digitalisation as the use of tools to convert analogue information into digital information. This broad definition comprises a wide range of our everyday ICT such as computers and smartphones. For instance, it includes punch cards to document absences at work, which were early digital devices at the workplace, which were used to contain digital data represented by the presence or absence of holes in predefined positions. From this perspective, digitalisation can refer to anything from the mere use of software such as Microsoft Word on a laptop or even a desktop computer, to food deliveries in the gig economy based on app-based management tools.

Importantly, the potential burden of technostress may be structured along a social gradient. In social science research on digital work and inequalities, there has been a growing interest in new forms of digital labour and precarious work emerging in the context of digitalisation. Social scientists have, for instance, examined the globalisation of digital labour and ethical implications of so-called click work and the gig economy. The growing literature on specific digital occupations shows that new and emerging digital economies entail new forms of precarious digital labour, resulting in specific risks for socioeconomically disadvantaged workers [3,4]. Thus, this systematic review asks: What is the role of socioeconomic position (SEP) in studies on associations between exposure to work-related techno-stressors and health or work outcomes?

### 1.1. The Technostress Model

The technostress model has been mostly applied with distinctions between the context of leisure and work activities. The most influential study defined technostress very broadly as an IT user’s experience of stress when using technologies [5]. Recent systematic reviews have provided a general overview of the technostress model [6] and of its associations with mental health [7]. As summarised in a comprehensive review of technostress studies, this has led to a repeated focus on a set of technology-related factors, i.e., as stimuli, events and demands perceived by individuals, which can cause technostress [6]. Five *techno-stressors* were introduced within the framework of the technostress model, which has remained largely unchanged [5,8]: techno-overload (technology forces workers to work more and faster); techno-invasion (invasion of private life due to technology that creates pressures of constant connectivity); techno-complexity (technology is complex, leading to a sense of lack with regard to computer skills); techno-insecurity (workers feel threatened about losing their jobs because of new technologies); techno-uncertainty (constant technological changes that may create stress for workers). 

The technostress model draws on Lazarus’ transaction theory of stress from organisational psychology [9] to highlight the conditions under which ICT exposure is experienced negatively. It emphasises that stress results from a combination of a workplace demand condition that causes the stress (stress creators or “stressors”) and the individual’s response to it (manifest adverse outcomes). By shaping the working conditions that frame an individual’s response, organisational factors may influence whether ICT use is perceived negatively. Recent studies that analyse digitalisation with respect to working conditions emphasise the importance of worker participation in the digitalisation process, which tends to be influenced by SEP, to mitigate negative effects on workers’ well-being [10].

In their systematic review, Berg-Beckhoff, Nielsen and Larsen [7] examine associations of ICT use with stress and burnout. They found a trend towards positive associations of technostress and burnout across different study designs. Furthermore, their review highlighted that only a few studies sufficiently specified organisational factors that influence ICT-related work processes. In a similar vein, a European Union foresight study [11] suggests that both psychosocial and organisational factors will become more important for occupational health, as digitalising work can drive changes that can increase the risk of workers’ stress (e.g., increased monitoring of workers, an assumption of 24/7 availability and the management of work and workers by algorithms).

### 1.2. Socioeconomic Position: A Social Determinant of Individual Techno-Stressors?

There are various variables to describe and measure socioeconomic conditions. In this review, we use “socioeconomic position” (SEP) to refer to the socially derived economic factors that influence what positions individuals or groups hold within the stratified structure of an organisation or society [12]. Individual-level indicators used in health research measure some types of individual resources such as education, income or wealth. Occupation-based indicators measure the position within an organisation and include job grade or position [13].

In general, higher SEP is associated with better self-rated health around the globe. Some evidence of a social gradient in health suggests that both education and income are more strongly associated with self-rated health than other sociodemographic variables [14]. There is robust evidence of a social gradient in the context of major chronic diseases and other health measures [15,16]. Work and employment conditions play a crucial role in attempts towards explaining this social gradient, given their primary impact on everyday life [17]. Laying the groundwork for later conceptual work within the emerging framework of social determinants of occupational health [18], the classic study by Ragu-Nathan, et al. [5] recommended analysing the effects of sociodemographic variables including education, age and experience on technostress [8]. 

Examining education as a social determinant of overall technostress, Ragu-Nathan, et al. [5] found that overall technostress decreased with increasing age and education. Tarafdar, et al. [19] also concluded that users with more formal education experienced less technostress. Krishnan [20] assessed the antecedent measures of age, gender, education, and computer confidence in an Indian sample of student alumni and found that after taking the Big Five personality traits into account, techno-stressors only showed significant associations with education (i.e., individuals with a higher level of education perceived techno-stressors more negatively). Of the Big Five personality traits, agreeableness was the strongest predictor of techno-stressors, followed by openness to experience. Despite such preliminary findings, studies since then have still focused less on the antecedents of technostress than on other topics [6] and studies implicitly generalise their findings without adequately addressing differences by SEP.

A second recent review characterised the structure of the research field in terms of the definition, symptoms and risk factors of both work-related and non-work-related technostress [6]. Concerning workplace technostress, the authors conclude that many more categories of workers need to be studied, especially those with different types of ICT use, and that studies should focus on single or specific techno-stressors. Yet, their review only occasionally distinguishes overall technostress and specific techno-stressors when describing the studies and reporting their findings.

Previous findings suggest that it may be important to individually examine associations between SEP and individual techno-stressors. With regard to techno-insecurity, recent studies found negative implications for workers’ psychological reactions [21] and job satisfaction [22]. The negative effect on job satisfaction is driven by low SEP workers, which are those carrying out routine-based tasks, and who are therefore more exposed to the risks of substitution at work [22]. A nationally representative German survey indicated that on average, 13% of workers reported high levels of techno-insecurity and showed that there is a clear social gradient, as 28% of workers with lower education expressed this expectation [23].

In a recent study of nurses that also aimed to validate a holistic model of technostress, techno-insecurity and techno-overload significantly influenced stress and job satisfaction [24]. The SEP factors influenced how the individual thinks about stressors and psychologically responds to the stressors. Another study of older German employees found techno-overload was negatively associated with mental health and work ability. However, the effects in these studies were of similar strength across all groups of SEP [25]. When differentiating individual techno-stressors, a study of public sector employees in Brazil did not identify differences related to the educational level of workers with regard to any of the techno-stressors [26]. These study findings are inconclusive, yet indicate that the effects of sociodemographic variables could potentially be sample-specific and specific to certain techno-stressors. 

Consequently, for this systematic review we differentiate the techno-stressors examined in the included studies in order to explore the role of socioeconomic factors with regard to individual techno-stressors. Moreover, a variety of measures of SEP have been applied to examine social and health inequalities [13]. Our review thus charts operationalisations and findings related to SEP in the field of work-related technostress to identify what is needed to better account for the socioeconomic implications of individual techno-stressors.

## 2. Methodology

We conducted the systematic review in accordance with the PRISMA statement guidelines [27] and registered the review protocol in the PROSPERO database in July 2020 (ID CRD42020199960).

### 2.1. Search Strategy and Study Selection

After initial dummy keyword searches to refine keywords, we conducted systematic literature searches for articles in June 2020. Given the multidisciplinary nature of technostress, we searched the following databases: PubMed and PubMed Central, Web of Science, Scopus, PsycInfo and PsycArticles. We identified duplicate records and removed them following scientifically validated steps for de-duplication with Endnote [28]. In addition, we manually searched reference lists in review articles to identify missing relevant studies.

To restrict our search to the work setting, we used a validated base search string for searches related to occupational health [29]. This validated search string was combined with technostress as a disease term (Appendix B shows the string for PubMed). We consulted an independent expert in occupational health research prior to the screening phase who confirmed the applicability of the search string.

As shown in Table 1, we defined eligibility criteria according to the population, intervention, comparator, outcome, study design (PICOS) scheme following pilot searches for studies on work-related technostress and initial assessments of relevant previous reviews [6,7,30,31]. To be eligible, the study population had to be working adults exposed to technostress related to ICT use for work purposes, which should be considered analytically distinct from non-work-related ICT use [6,31]. We searched for technostress as a keyword as studies merely assessing the use of ICT in general were considered to be outside the scope of this study unless they explicitly drew links to the technostress model. We included studies that addressed a person’s mental health and work outcomes as both are frequently studied in association with technostress [32]. Finally, eligible publications had to be peer-reviewed, available in English and report original data from empirical studies.

Two reviewers (P.B. and K.R.) independently screened the identified articles based on titles and abstract, then conducted the full-text screening using Rayyan (http://rayyan.qcri.org accessed on 3 December 2020), a free web and mobile app, that helps expedite the initial screening of abstracts and titles using a process of semi-automation. If at any stage of study selection discrepancies arose that could not be resolved by consensus between the two reviewers, a third reviewer (S.V.M.) was consulted to reach a final decision. Finally, references of reviews were checked for potentially relevant articles.

### 2.2. Data Collection and Quality Appraisal 

Two reviewers (P.B. and K.R.) defined the key themes for data extraction and one reviewer (P.B.) then extracted the data. We first piloted a data extraction form to identify relevant data categories and then extracted data on:

- study population (sample description, country of data collection, participant characteristics and occupational setting),
- study design,
- details of the exposure, i.e., measure of technostress,
- outcome measures,
- indicators of SEP (incl. income, education, socioeconomic status, job position, etc.),
- results regarding differences by SEP,
- confounders.

To assess the risk of bias of the included studies (P.B.), the Newcastle–Ottawa scale (NOS) adapted for cross-sectional studies was used [33]. The NOS has been previously applied in systematic reviews of technostress [6,7]. The NOS consists of three sections—selection (max. five stars), comparability (max. two stars), and outcome (max. three stars)—and scores papers on a scale of 0 to 10 stars in total. The risk of bias can be grouped into high risk (score 0–3), medium risk (score 4–6), and low risk (score 7–10), i.e., high scores denote low risk of bias. Whenever necessary, uncertainties regarding extracted data and the NOS were resolved by discussion (P.B. and K.R.).

## 3. Results

**Figure 1:**
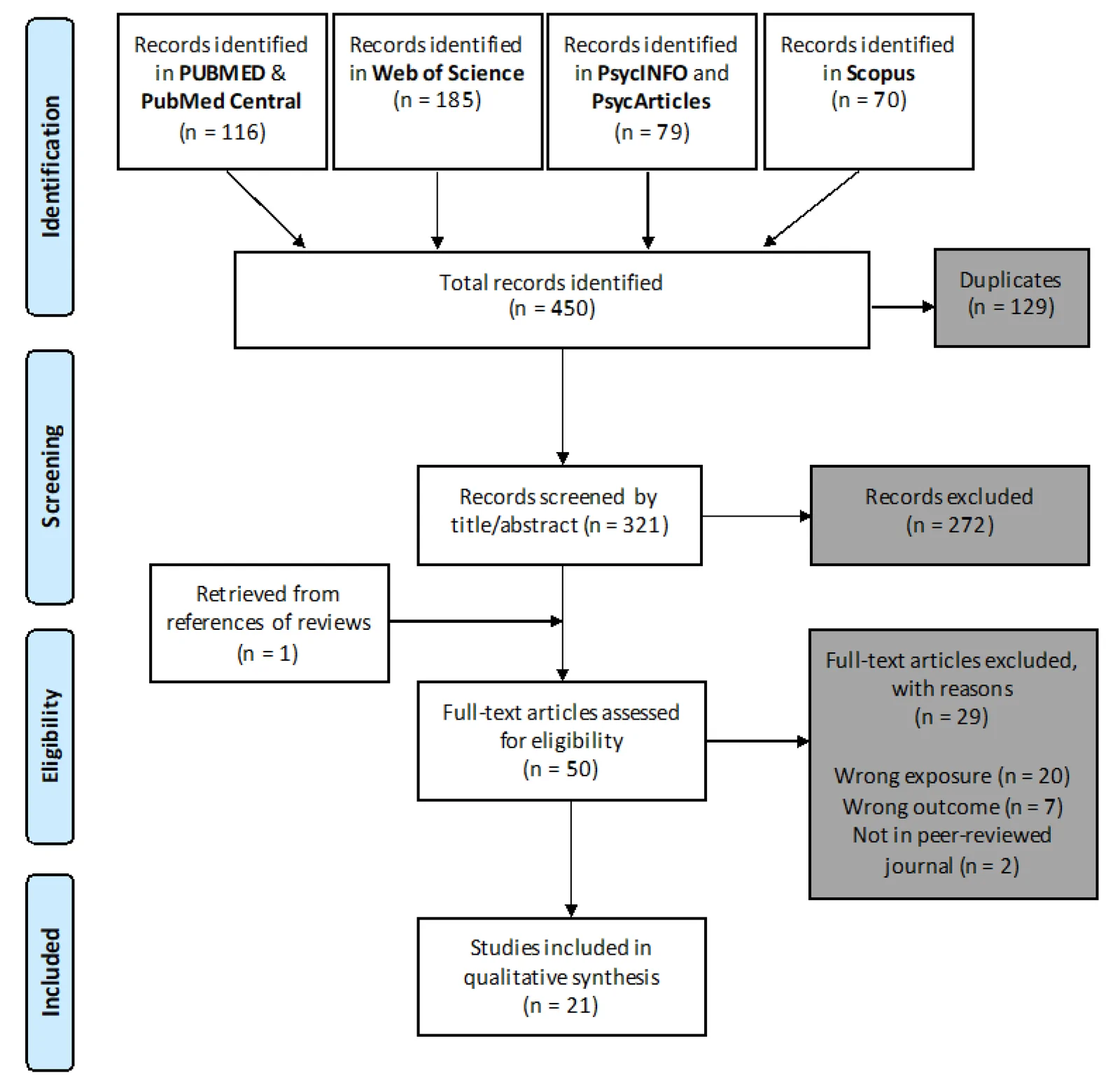
Preferred reporting items for systematic reviews and meta-analyses (PRISMA) flow diagram depicting the number of reports screened and included in this systematic review.

The preferred reporting items for systematic reviews and meta-analyses (PRISMA) flow chart in Figure 1 shows the study selection process. Our search in four multidisciplinary electronic databases yielded 450 records in total. After removing duplicates, we screened 321 articles based on titles and abstract. One additional article was identified and retrieved from the references of review articles. Most studies were excluded because they examined exposure or outcomes variables that should be considered analytically distinct from our focus on assessing the influence of digitalised work on health and wellbeing at the workplace. For example, we excluded studies that did not measure a health or personal work outcome (e.g., job burnout and job satisfaction) and studies that examined technostress as an outcome variable rather than an exposure. As a result, 50 articles were then included in the full-text screening.

During the full-text screening, we excluded 20 articles because they measured a different exposure, seven because they measured a different outcome, and two because they were not published in peer-review journals. Some excluded studies measured an exposure variable that did not match with the technostress model (e.g., information overload that is operationalised differently from techno-overload).

A complete summary of the 21 articles included in the systematic review is available as Appendix A. The included studies displayed considerable heterogeneity in choice and analyses of ICT exposure and outcome measures. Because the sampled occupations, exposure measures, and reported outcome measures varied markedly, our review focuses on describing and assessing the studies, their results, their applicability, and their limitations through a qualitative synthesis of the following: quality appraisal (Section 3.1) study characteristics (Section 3.2), the role of SEP as effect modifier between technostress and health or work outcomes (Section 3.3) and socioeconomic sampling bias (Section 3.4).

### 3.1. Quality Appraisal

We assessed the methodological quality of the included studies with the Newcastle–Ottawa scale (NOS), which has adapted for cross-sectional studies [33]. Overall, nine studies demonstrate low risk, 10 studies a medium risk, and two studies a high risk of bias (see Table 3). NOS scores ranged from a minimum of 3/10 to a maximum of 9/10 with higher score representing better quality. The median score is 6. Only two studies, which were based on the same nationally representative dataset from Sweden, reached the best score of 9 [34,35], followed by two studies based on simple randomised samples [36,37].

Overall, 19 studies were cross-sectional and only two had a longitudinal study design. 16 of the 21 studies were based on convenience samples and reported a large range of sample sizes, mostly with low response rates. Of the five randomised study samples, three reported satisfactory response rates, while studies based on commercial online panels did not report response rates at all. The other two were simple randomised samples that did not enable comparability between respondents and non-respondents regarding potential confounders. Seven studies did not describe any confounders, while the rest considered at least one potential confounder.

### 3.2. Study Characteristics

The majority of studies have been conducted in the USA with a total of six, followed by three in South Korea (Table 2). The 21 included studies covered multiple disciplines, with the most being situated in information systems, i.e., ICT-oriented business research (14), followed by psychology (4), public health (2) and social sciences (1). A large majority of the 16 studies were based on convenience samples collected in various organisations. Sample sizes ranged from 152 to 608 employees. The reported occupational sample settings suggest there could be a socioeconomic sampling bias as several studies only sampled (senior) managers and executives [38,39,40,41], academics [42], employees in the IT industry [43,44] or white collar employees [45,46]. Occupational sample settings were also selected due to a typically higher frequency of ICT use [47,48].

The other five studies were based on randomised samples with sample sizes ranging from 374 to 14,757 employees. Only two of these randomised samples were drawn from national employment registers and therefore nationally representative of the working population [34,35]. The other three were drawn from commercial online panels with potential limitations to generalisability [36,37,44]. The response rates ranged from 16%–89%, while seven studies did not report any response rate and two authors provided these data upon request [36,44]. Age and gender distributions varied depending on the corresponding occupational sample settings. In most studies, samples were not equally distributed by gender and age, but which groups were overrepresented varied greatly between studies. For instance, whereas in the study by Tarafdar, Tu, Ragu-Nathan and Ragu-Nathan [8] men were underrepresented (17%), in a study by Srivastava, Chandra and Shirish [39] men were overrepresented (76%).The proportion of men in the samples ranged from 17% to 82%. A Swedish nationally representative study reported that younger people were underrepresented [35].

### 3.3. The Role of SEP in Effects of Work-Related Technostress on Health and Work Outcomes

Table 3 showed that 18 of the 21 included studies (86%) collected data on socioeconomic position: education (eight), job position (five), two studies measured SES (two) and both educational as well as job position (three). Three studies did not report any collection of SEP data. Overall, seven studies treated SEP as a potential confounder and two studies performed stratification or subgroup analyses to assess differences by SEP.

Regarding outcomes, 14 studies (66%) examined at least one of five different health outcomes: strain and stress (4), self-rated health (3), negative emotion and anxiety (3), burnout (2), and work exhaustion (2). Twelve (57%) studies examined at least one of three different work outcomes, i.e., job satisfaction (6), productivity or performance (5), and work engagement (2).

Regarding exposure to technostress, six studies measured the exposure to all subconstructs of technostress. In studies that did not use terminology from the technostress model, we compared the variables and survey items to the technostress model and its subconstructs, i.e., techno-stressors, and categorised accordingly. Eight of the 21 included studies measured the technostress model with all subconstructs as the exposure (see Appendix A). The most commonly studied subconstructs are techno-overload (included in 17 of 21 studies), followed by techno-invasion (16 studies) and techno-insecurity (15 studies). It should be noted that the terminology and measurement of SEP as well as technostress exposure has not been consistent across studies.

#### 3.3.1. Socioeconomic Position Operationalised as Education

The study by Ragu-Nathan, Tarafdar, Ragu-Nathan and Tu [5] that was pivotal in introducing the technostress model reported a negative association between education. Their study achieved an NOS score of 8, noting that individual differences were only tested on technostress overall, not individual techno-stressors, and that later studies should explore the role of sociodemographic variables. In longitudinal analyses of a simple randomised sample from a commercial online panel, Goetz and Boehm [36] showed a significant direct and negative effect of techno-insecurity on self-rated health measured at a later point in time. However, this recent study reported that, contrary to expectations, techno-insecurity was not correlated with educational level [36]. This study achieved an NOS score of 8. Tarafdar, et al. [49] found education to be positively associated with the work outcome productivity but did not report differences by SEP in the effects of technostress and achieved a medium NOS score of 4.

#### 3.3.2. Socioeconomic Position Operationalised as Job Position

Having operationalised SEP as job position (senior executives vs. middle managers), Vayre and Vonthron [41] found no significant differences between both groups. With regard to work engagement, executive officers, CEOs, or senior executives were more dedicated to their work than middle managers. This study achieved a NOS score of 7. In preliminary descriptive analyses of the influence of job position, Khedhaouria and Cucchi [38] found that respondents in senior management positions intensively used ICT for their professional tasks and that they were more exposed to job strain than other employees. However, their study did not report further associations after excluding the subgroup of employees from their further analyses and reached a NOS score of 6. Finally, a study in the aviation industry found that the negative association between techno-stressors and productivity was stronger when workers were equity-sensitive [50]. Moreover, results indicated that respondents who occupied senior management positions used ICT more intensively for their professional tasks than employees, but data for this claim were not presented. The study reached a NOS score of 5.

#### 3.3.3. Socioeconomic Position

Two studies based on Swedish representative data [34,35] achieved the highest NOS scores of 9 among included studies. Both performed stratified analyses that found a higher prevalence of technostress, operationalised as ICT demands, among employees in higher SEPs. Although the earlier study found negative associations between technostress and self-rated health, the strength of effect on self-rated health did not vary significantly in different SEP [35].

A follow-up study based on longitudinal data again found SEP differences in technostress at work based on descriptive analyses [34]. Repeated exposure to high levels of technostress (measured at T1 and T2) was more common among participants with high SEP (40.0%), followed by participants with intermediate SEP (35.5%) and low SEP (12.5%). In further analyses, the SEP-stratified crude analysis showed that repeated exposure to high technostress at work was associated with increased risk of developing worse self-rated health among participants with high SEP, followed by participants with low SEP. But when the analyses were additionally adjusted for age, sex, health behaviours, body mass index (BMI), job strain and social support, the risk among participants with low SEP was slightly increased and attenuated, while it was no longer statistically significant among participants with high SEP. More importantly, a test for statistical interaction between technostress and SEP in the total study population was not statistically significant in any of the regression models.

### 3.4. Socioeconomic Sampling Bias in Subsample of Included Studies

We compared the study samples to representative data on the general working population to assess the extent of socioeconomic sampling bias (Table 4). To this end, we identified a subsample of 13 studies that reported descriptive SEP data comprising educational attainment. Eleven studies reported descriptive SEP data operationalised as education and two as SEP from which we extracted educational attainment. Because several studies only reported the percentage of university graduates, we compared the percentage of university graduates reported by studies in this subsample to national reference data on educational attainment collected and provided by the World Bank (https://data.worldbank.org/indicator/SE.TER.CUAT.BA.ZS, accessed on 3 December 2020). For this comparison, we used data from the year closest to the year of data collection in the study.

Overall, our assessment shows that of the 13 studies in this subsample, 11 studies collected data from workers with higher SEP compared to the general population. The only two studies that reported an SEP distribution that coincides with national data were based on a Swedish nationally representative sample [34,35]. Both studies are based on samples drawn from the same national study data and reported 22%–23% university graduates, which matches the range reported by the World Bank between 2013 and 2014.

Among the 11 studies with a socioeconomic sampling bias, the percentage gap between university graduates in the respective study samples and nationally representative data from the World Bank ranged from 6% to 87.5% (median: 36%). Convenience samples displayed a bias towards higher SEP than the national average. In all three studies based on simple randomised samples from commercial online panels, university graduates were overrepresented. Moreover, all the authors who can be considered foundational to the technostress model (authors underlined in Table 4), according to scientometric analyses of citations in the field of technostress [51] display a socioeconomic sampling bias.

**Table 4:** Socioeconomic sampling bias in a subsample (sorted by % gap in university graduates).

First Author and Year	Sample Type	Country Data	University Graduates (Study)	University Graduates (National) **	% Gap	SEP Bias
Stadin et al., 2016	randomised (nationally representative)	Sweden	22% ***	23% (2014)	−1.0%	mixed SEP
Stadin et al., 2019	randomised (nationally representative)	Sweden	23% ***	22% (2013)	1.0%	mixed SEP
Goetz and Boehm 2020	randomised (commercial online panel)	Germany	31%	25% (2018)	6.0%	high SEP
Day et al.,* 2012 *	convenience	Canada	53%	26% (2016)	27.0%	high SEP
Tarafdar et al., 2007 *	convenience	USA	60%	32% (2013)	28.0%	high SEP
Tarafdar et al., 2011 *	convenience	USA	60%	32% (2013)	28.0%	high SEP
Tarafdar et al., 2015 *	convenience	UK	70%	34% (2017)	36.0%	high SEP
Suh and Lee 2017	convenience	South Korea	65%	29% (2015)	36.0%	high SEP
Ayyagari et al., 2011 *	randomised (commercial online panel)	USA	75%	32% (2013)	43.0%	high SEP
Ragu-Nathan et al., 2008 *	convenience	USA	77%	32% (2013)	45.0%	high SEP
Lee 2016	convenience	South Korea	76%	29% (2015)	47.0%	high SEP
Al-Ansari and Alshare 2019	convenience	Qatar	70%	19% (2017)	51.0%	high SEP
Wu et al., 2020	randomised (commercial online panel)	China	91%	3.5% (2010)	87.5%	high SEP

* Authors central in scientometric analyses of the technostress field are highlighted [52]. ** Comparison data on educational attainment were collected from the World Bank data repository at https://data.worldbank.org/indicator/SE.TER.CUAT.BA.ZS (accessed on 3 December 2020). *** As the SEP variable reported was SES/SEP as opposed to education, we converted the proportion of high SEP into university graduates.

## 4. Discussion

### 4.1. The Role of SEP and Socioeconomic Sampling Bias in Technostress Studies

Our review compared the socioeconomic distributions and analyses of SEP in research on work-related exposure to technostress to assess whether specific techno-stressors are distributed differently according to SEP. Our assessment shows that in a subsample of 13 studies, 11 studies collected data from workers with higher SEP compared to the general population, thus constituting a socioeconomic sampling bias. This socioeconomic sampling bias is, in part, an expression of the more general issue that behavioural studies routinely publish broad claims based on samples drawn entirely from Western, educated, industrialised, rich, and democratic (WEIRD) study populations [52,53]. Although samples in technostress studies show some variation with regard to geographic and political contexts, they have been focused on overly educated and rich populations and professions.

The only two studies that reported an SEP distribution that coincides with national data were based on the same nationally representative study in Sweden [34,35]. Even these studies reported that workers with lower SEP were less likely to respond, hence, the increased risks of selection biases in convenience samples. Studies based on convenience samples did not adequately highlight the limitations due to socioeconomic sampling biases. Furthermore, studies based on simple randomised samples from commercial online panels displayed a bias to higher SEP, apart from one study that came close to being representative regarding SEP [36]. This issue has been reported in earlier methodological assessments of commercial online panels [54,55].

Despite such limitations, several studies with high NOS scores analysed differences by SEP, albeit with partially conflicting results [5,34,35,36]. The foundational study in establishing the technostress model reported a negative association between education and technostress [5]. In contrast, Goetz and Boehm [36] unexpectedly did not find differences in technological insecurity by educational level. Their study, despite being based on a commercial online panel, suffered from a relatively low socioeconomic sampling bias, but did not report further analyses of SEP. Similar representative studies have found higher levels of education to be associated with increased ICT use and decreased techno-insecurity [23].

The representative Swedish studies on overall technostress showed no signs of socioeconomic sampling bias, but were also inconclusive. Both concluded that in terms of prevalence, high SEP workers were more affected by technostress and that consistently high technostress over time was most common among participants with high SEP [34,35]. However, a test for statistical interaction between techno-overload and SEP in the total study population was not statistically significant in any of the regression models applied in the follow-up study.

Techno-invasion appeared more relevant to occupations with a high SEP. Vayre and Vonthron [41] try to justify the high SEP in their sample based on findings that suggested frequent ICT users at work are often employees with higher degrees, in middle management or executive jobs [56,57]. They explain that the increase in work hours associated with the growth of ICTs, as well as the use of ICT outside working hours particularly affects managers [58]. Thus, there is some evidence that techno-overload and techno-invasion may be more prevalent among high SEP occupations. As there are only some conflicting findings regarding techno-insecurity, there is a general lack of evidence regarding low SEP occupations.

To date, a sizable number of studies showed signs of falling short of enough variance in SEP for anything more than descriptive analyses [41,59]. For instance, Wang and Li’s (2019) measure of grade levels is vague and conceals that their study comprises only university teachers who represent higher SEP positions. Overall, when a measure of SEP was collected, it was in most studies only treated as a confounder in analyses without considering potential subgroup effects. Thus, more analyses of representative samples stratified by SEP are needed to explore how SEP influences technostress.

With a high degree of conclusiveness, the findings in the included studies indicate that the effects of sociodemographic variables are sample-specific and context-dependent. Yet, a substantial proportion of data on technostress were collected in occupations with extremely high SEP and disproportionately often in the IT sector. This sampling bias could result due to the relative ease of establishing a study cooperation with the usually well-regarded, often hyped, IT sector and thereby gaining access to participants and occupational data [60]. This caveat may affect much of science–industry collaboration, in particular for research on (new) technologies due to its particularly privileged, pioneering and hype-prone image in the context of work [1,61].

### 4.2. A research Agenda to Conceptualise Socioeconomic Differences in the Context of Technostress

Although different exposures and subconstructs have been discussed under the rubric of technostress [6,30], doubts have been raised as to whether they appropriately capture cross-cultural [62] and socioeconomic differences [34]. As a case in point, techno-invasion and work–family conflict may be more applicable to higher SEP [41], an assumption for which we found some evidence. Our systematic review thus echoes recent studies that have begun to call for more theory development and emphasise the importance of social context in technostress research [50,63]. Suggestions range from a taxonomy of ICT [37] to context-specific typologies of relations between types of users, technological artefacts and work settings [63]. Future research should also promote conceptual research on individual techno-stressors and social stratification to effectively differentiate the implications of new forms of digital labour.

If future studies were representative of the general working population, this would help to avoid potential biases in convenience and commercial samples. Conversely, the field must encourage and facilitate more diligent reporting of limited generalisability. For instance, it should be noted that the foundational study of the ICT demands scale [45] was based on a sample of higher SEP workers and thus validated in a study sample biased towards high SEP without highlighting this as a limitation. Fifty-three percent held a university degree, with an additional 34.5% holding a graduate or professional degree. Hence, a limitation of the ICT demands scale is that it may be more sensitive to technostress that is specific to white collar office work.

Furthermore, a striking majority of studies examined links between technostress and inequalities by operationalising SEP with the variable education, despite a wide variety of indicators of SEP to examine health inequalities [13]. There is some evidence that both education and income are more strongly associated with self-rated health than other sociodemographic variables [14]. Yet, none of the studies included in our review reported analyses based on income data. While income can be a particularly valid measure of status in the context of work [12,64], evidence suggests that personal income is a sensitive question that leads to increased non-response [65]. Nonetheless, income data could help to ensure greater variance and potential for statistical inference. Consequently, there is a need to explore the relative advantages and disadvantages of different measures of SEP, specifically in the context of ICT at work.

As a consequence of techno-stressors quickly becoming dated in such a dynamic field of research, conceptual work is needed to regularly expand the technostress model to include new phenomena. A narrow focus on techno-stressors in the established technostress model may reinforce social and health inequalities in as far as there are other factors that are more strongly linked to socioeconomic differences. Yet, there is a lack of evidence to indicate which subconstructs of technostress and outcomes are more strongly linked to socioeconomic differences, which may reinforce social and health inequalities. For instance, adapting the technostress model to encompass workplace surveillance, performance monitoring, task monotony and algorithmic management [66], would uncover how ICT shapes the working conditions of socioeconomically more marginalised groups.

Overall, future conceptual work is needed to develop a model of work-related ICT exposure that is also sensitive to socioeconomic differences. This would not only include a taxonomy of ICTs proposed by Ayyagari, Grover and Purvis [37]. Rather, it must aim for context-specific typologies that specify relations between types of users, technological artefacts and settings of use [64]. Such a model could address the role of the social and ethical implications of ICT in the context of technostress by giving social matters and struggles more visibility [1,67,68].

Finally, previous applications of tools such as the NOS to assess the risk of bias have not sufficiently highlighted socioeconomic sampling biases. Much like feminist quality appraisal tools that aim to highlight gender biases [69], developing socioeconomically sensitive risk of bias assessments is necessary to shed light on hidden socioeconomic sampling biases. Such a tool could be based on a comparison of study data on education to national data as shown in this review. The issue that study results have been discussed without adequately raising limitations regarding generalisability is not only problematic in the context of research. From the perspective of practitioners and other stakeholders, it may be a challenge to apply research tools with sufficient methodological know-how and gain a full picture in order to develop workplace interventions. If practitioners aim to implement interventions based on a model of technostress that has been developed without carefully highlighted socioeconomic blind-spots, it can become inherently exclusionary and further marginalise certain social groups.

### 4.3. Limitations

The interpretation of our findings is subject to several limitations. First, many studies may not use the term “technostress” when researching ICT use at work. The search term “technostress” may be less effective to retrieve older studies and research in the social sciences given that the concept has only relatively recently gained more traction across scientific disciplines. Second, we had to limit our search to quantitative study designs to enhance comparability of studies in terms of outcomes and, particularly, measures of SEP. Notwithstanding this, our assessment of the extent of socioeconomic sampling bias (see Table 4) was only based on a subsample of 13 of the 21 included studies due to the limited comparability of SEP measures. Third, both technostress exposure and outcome measures were limited to self-reported measures. Further systematic reviews could cover related topics with regard to biological measures of health as has been described in relation to technostress [70,71].

## 5. Conclusions

Our systematic review reassesses what has too uncritically been described as a measurement pluralism in the field of technostress [30]. It shows the high heterogeneity of measures of technostress, SEP as well as outcomes in the context of the technostress model. The choice of SEP indicators and the overall limited knowledge of the role of SEP reflect methodological issues in the reviewed studies. We argue that socioeconomic sampling biases in research on work-related technostress has limited the amount of evidence regarding the socioeconomic implications of technostress at work. Due to potential limitations of the study samples used to study work-related technostress, the generalisability of sample-specific and context-dependent effects depending on work setting and SEP need to be identified.

We reviewed the role of SEP within the field of technostress and found—in almost all sampled studies—the existence of significant socioeconomic sampling bias. This often-hidden socioeconomic sampling bias has remained insufficiently explored and discussed in the context of studies based on the technostress model. Thus, the technostress model has been developed based on study samples that comprise exclusively high SEP occupations. Even the studies that have become foundational to technostress research are limited in scope and suffer from a socioeconomic sampling bias towards white collar workers and occupations. Consequently, there are very limited empirical findings regarding the role of SEP in work-related technostress, which are at least in part a result of a hidden socioeconomic sampling bias.

In general, stakeholders involved with digitalisation in work settings would benefit from approaching it as a dynamic process of both social and technological change. In other words, we need to attend to social and organisational factors that influence how digital devices are implemented, who can participate in their implementation and which techno-stressors are experienced. Future research should assess SEP as an effect modifier on the level of separate techno-stressors. To this end, studies must report analyses of individual techno-stressors separately to assess differences by techno-stressors, instead of opting to merely report technostress as a composite score. Compared to previous literature reviews, our review provides additional detail and data by distinguishing associations both on the level of overall technostress as well as the level of techno-stressors. We found that various studies discuss technostress as the overall concept of exposure, although they only measured a selection of subconstructs from the original model without distinguishing overall technostress and specific techno-stressors. This lack of consistency further conceals the socioeconomic sampling biases that may be inherent to subconstructs of technostress.

## Figures and Tables

**Table 1:** Eligibility criteria: PICOS (population, intervention, comparator, outcome, study design).

	Inclusion Criteria	Exclusion Criteria
Population	Working adults.	Non-work-related samples and workers below the age of 18.
Intervention/ Exposure	Technostress at work.	Does not measure work-related exposure to at least one techno-stressor.
Comparator	Any comparator including no intervention.	
Outcome	Mental health or work outcomes (e.g., job burnout and job satisfaction)	Does not measure mental health or personal work outcomes. Measures technostress and ICT use as outcomes.
Study design	Observational studies with a cross-sectional or, preferably, longitudinal design. Quantitative studies.	Non-empirical studies (commentaries, policy briefs, review articles). Qualitative studies. Unclear study design.
Others		No English translation available. Not published in peer-review academic journal before 8 June 2020.

**Table 2:** Occupational setting and sample.

First Author and Year	Discipline	Sample Type	Country Data	N (RR)^a^	Occupational Sample Setting	Gender (% Men)	Age
Al-Ansari and Alshare 2019	Information Systems	convenience	Qatar	410 (-)	work-related ICT users with daily use	33%	<31 (37%); 31–40 (32%); >41 (31%)
Alam 2016	Information Systems	convenience	Pakistan	203 (68%)	aviation industry	82%	Not reported
Ayyagari et al., 2011	Information Systems	randomised (commercial online panel)	USA	661 (-)	employees with access to internet-enabled ICT	52%	Mean: 49 Median 52
Day et al., 2012	psychology	convenience	Canada	258 (-)	mixed sample of occupations (e.g., engineers, accountants, management, psychologist, IT specialist)	47%	Mean: 35
Florkowski 2019	Information Systems	convenience	USA	177 (22%)	169 companies with human resources technologies	not reported	Not reported
Gaudioso et al., 2017	psychology	convenience	USA	242 (16%)	1 large government-related organisation	28%	Modal age bracket: 45–54 years
Goetz and Boehm 2020	Information Systems	randomised (commercial online panel)	Germany	8019 (59%)	employees with access to internet-enabled ICT	51%	Mean: 44.3 (range: 18–77)
Jena 2015	Information Systems	convenience	India	216 (54%)	academics in multiple higher education organisations	54%	>35 (53%); <35 (47%)
Khedhaouria and Cucchi 2019	Information Systems	convenience	France	465 (46%)	senior managers in various economic sectors (industry, commerce and services)	50%	Mean: 39 (range: 24–62)
Kim et al., 2015	social sciences	convenience	South Korea	210 (-)	companies with mobile enterprise system	70%	>40 (60%)
Lee 2016	psychology	convenience	South Korea	222 (-)	3 companies	81%	20–29 (1.7%), 30–34 (17.6%), 35–39 (17.1%), 40–44 (35.1%), 45–59 (22.5%)
Ragu-Nathan et al., 2008	Information Systems	convenience	USA, India	608 (89%)	white collar employees in 5 organisations (government, manufacturing and financial)	63%	below 45 (>50%)
Srivastava et al., 2015	Information Systems	convenience	international	152 (22%)	senior managers and executives	76%	Mean 37.96 (SD: 6.73)
Stadin et al., 2016	public health	randomised (nationally representative)	Sweden	14,757 (53%)	national representative sample	43%	Range: 20–75
Stadin et al., 2019	public health	randomised (nationally representative)	Sweden	4468 (61%)	national representative sample	43%	Mean: 47.3 Most 40–59
Suh and Lee 2017	Information Systems	convenience	South Korea	258 (86%)	2 global IT companies with telework programs	57%	Not reported
Tarafdar et al., 2015	Information Systems	convenience	UK	237 (47%)	sales professionals from 3 companies	66%	Not reported
Tarafdar et al., 2007	Information Systems	convenience	USA	233 (73%)	2 public sector organisations	17%	Not reported
Tarafdar et al., 2011	Information Systems	convenience	USA	233 (73%)	2 public-sector organisations (middle-management positions)	17%	Range: 26–56
Vayre and Vonthron 2019	psychology	convenience	France	502 (67%)	senior managers and executives	50%	Mean: 40
Wu et al. 2020	Information Systems	randomised (commercial online panel)	China	374 (62%)	managers and general staff in manufacturing and IT industries	42%	26–35 (75%)

^a^ RR response rate, “-” indicates RR was not reported or could not be calculated due to study design.

**Table 3:** Differences by SEP (sorted by NOS score).

First Author and Year	Techno-Stressor(s)	Outcome	SEP Measure	SEP Distribution	Findings Regarding SEP ^a^	NOS Score ^b^
Stadin et al., 2016	Technostress (operationalised as ICT demands)	self-rated health	socioeconomic position (SEP)	23.0% high 48.4% intermediate 28.6% low	- Techno-overload was most prevalent among participants with high SEP (59.8%), followed by participants with intermediate SEP (54.9 %) and low SEP (29.1 %). - The strength of effect of technostress on self-rated health did not show statistically significant differences by SEP. - When the analysis was stratified by SEP, the association was somewhat stronger between ICT demands and suboptimal self-rated health among participants with intermediate SEP (OR 1.62 (CI 1.42–1.86)), followed by participants with low SES (OR 1.39 (CI 1.18–1.63)) and high SEP ((OR 1.36 (CI 1.10–1.68)), adjusted for age, sex, lifestyle and body mass index (BMI)). Similar and consistent patterns were observed in the crude and all adjusted analyses. However, test for statistical interaction between ICT demands and SEP was not statistically significant in any of those models. - People with low SEP, men, and younger people were less likely to respond.	9
Stadin et al., 2019	Technostress (operationalised as ICT demands)	self-rated health	socioeconomic position (SEP)	23.2% high 47.4% intermediate 29.4% low	- Repeated exposure to high techno-overload was most prevalent among participants with high (40%), followed by participants with intermediate (35.5%) and low SEP (12.5%). - The SEP-stratified crude analysis showed that repeated exposure to high ICT demands at work was associated with increased risk of developing suboptimal self-rated health among participants with high SEP (OR 1.76 (CI 1.10–2.84)), followed by participants with low SEP (OR 1.61 (CI 1.03–2.52)). When the analyses were additionally adjusted for age, sex, health behaviours, BMI, job strain and social support, the OR among participants with low SEP was slightly increased (OR 1.67 (CI 1.04–2.66)) but attenuated and was not statistically significant among participants with high SEP (OR 1.56 (CI 0.94–2.60)). The risk was lower and not significant among participants with intermediate SEP either in the crude analysis (OR 1.24 (CI 0.91–1.69)) or in the analysis adjusted for age, sex, health behaviours, BMI, job strain and social support (OR 1.07 (CI 0.77–1.49)). - A test for statistical interaction between ICT demands at work and SEP in the total study population, was not statistically significant in any of the regression models. - People with low SEP, men, and younger people were less likely to respond.	9
Goetz and Boehm 2020	Techno-Insecurity	self-rated health	education, job position	31.4% university degree 21.0% higher education entrance qualification 39.3% secondary school leaving certificate 8.4% lower vocational background	- Significant direct and negative effect of techno-insecurity assessed at time 1 on general health at time 2 (*B* = −0.27, *p <* 0.001; *F* = 81.47, *df* = 3, *p <* 0.01). - Techno-insecurity was not correlated with educational level (*M* = 4.75; *SD* = 1.00; *r* = −0.00; n.s.).	8
Ragu-Nathan et al., 2008	Technostress	job satisfaction	education	18% Master’s degree 59% Bachelor’s degree 10% Two-year college 7% High school 6% other	- technostress decreased as age, education, and computer confidence increased (individual differences were only tested on technostress overall, not individual technostress creators).	8
Ayyagari et al., 2011	Techno-Overload, Techno-Insecurity, Techno-Invasion	strain	education	22.3% Postgraduate 11.0% Graduate School 42.3% Graduated College 17.0% Some College 7.2% High School	-not specified or reported	8
Srivastava et al., 2015	Technostress	burnout, work engagement	-	-	-not specified or reported	7
Day et al., 2012	Technostress (operationalised as ICT demands)	stress, strain	education	53% university degree 34.5% graduate or professional degree	-sample characteristics	7
Vayre and Vonthron 2019	Techno-Invasion	work engagement	job position	57.6% CEO, senior executive, executive officer 42.4% Middle manager	-There were no significant differences between the four categories of daily work-related uses according to individual characteristics. -work engagement: executive officers, CEOs, or senior executives were more dedicated to their work than middle managers (*M* = 15.92; *SD* = 3.14 vs. *M* = 14.78; *SD* = 3.75).	7
Gaudioso et al., 2017	Techno-Overload, Techno-Invasion	work exhaustion	-	-	-not specified or reported	7
Kim et al., 2015	Techno-Overload, Techno-Invasion, Techno-Insecurity, Techno-Complexity	job satisfaction, work exhaustion	job position	7.6% Senior manager 31.4% General manager 17.6% Manager 21.9% Assistant manager 21.5% Staff	-sample characteristics	6
Khedhaouria and Cucchi 2019	Techno-Overload, Techno-Invasion, Techno-Insecurity	burnout	job position	Senior managers vs. employees	-Respondents who occupied senior management positions intensively used ICT for their professional tasks and were more exposed to job strain than employees.	6
Wu et al., 2020	Techno-Invasion	negative emotion	education, job position	91.4% university degree 49% mid-level management 18% general staff	-sample characteristics	6
Alam 2016	Techno-Overload, Techno-Complexity, Techno-Uncertainty	productivity	job position	90 pilots 113 supporting/ maintenance crew of varying age, experience and job categories	-not specified or reported	5
Tarafdar et al., 2015	Technostress	productivity	education	70% university degree	-Education levels were positively related with productivity.	5
Suh and Lee 2017	Techno-Overload, Techno-Invasion, Techno-Insecurity	job satisfaction, strain	education	15% above postgraduate 20% postgraduate 65% college	-sample characteristics	5
Lee 2016	Techno-Overload	negative emotion	education, job position	education: 76.1% higher education 23.9% high school degree Job position: 64.9% middle managers, 29.7% were assistant managers 5.4% entry level	-sample characteristics	4
Florkowski 2019	Techno-Insecurity	job satisfaction, stress	job position	-	-not specified or reported	4
Tarafdar et al., 2007	Technostress	productivity	education	14% Master’s 46% Bachelor’s 16% High school 19% Two-year college 5% others	-not specified or reported	4
Tarafdar et al., 2011	Technostress	productivity	education	14% Master’s 46% Bachelor’s 16% High school 19% Two-year college 5% others	-not specified or reported	4
Al-Ansari and Alshare 2019	Technostress	job satisfaction, organisational commitment, perceived performance	education	70% Bachelor’s degree	-not specified or reported	3
Jena 2015	Techno-Overload, Techno-Invasion	job satisfaction, organisational commitment, perceived performance, negative emotion	-	not reported	-not specified or reported	3

^a^ N.s. indicates “not significant”; ^b^ NOS score out of a maximum of 10 points.

## Data Availability

No new data were created or analyzed in this study. Data sharing is not applicable to this article.

## Appendix A

**Table A1:** List of included studies.

First Author and Year	Study Title	Journal Discipline	Techno- Overload	Techno- Invasion	Techno- Insecurity	Techno- Complexity	Techno- Uncertainty
Al-Ansari and Alshare [48]	The Impact of Technostress Components on the Employees Satisfaction and Perceived Performance: The Case of Qatar	Information Systems	x	x	x	x	x
Alam [51]	Techno-stress and productivity: Survey evidence from the aviation industry	Information Systems	x			x	x
Ayyagari, Grover and Purvis [37]	Technostress: Technological antecedents and implications.	Information Systems	x	x	x		
Day, Paquet, Scott and Hambley [45]	Perceived Information and Communication Technology (ICT) Demands on Employee Outcomes: The Moderating Effect of Organisational ICT Support.	psychology	x				
Florkowski [46]	HR technologies and HR-staff technostress: an unavoidable or combatable effect?	Information Systems			x		
Gaudioso, et al. [72]	The mediating roles of strain facets and coping strategies in translating techno-stressors into adverse job outcomes.	psychology	x	x			
Goetz and Boehm [36]	Am I outdated? The role of strengths use support and friendship opportunities for coping with technological insecurity.	Information Systems			x		
Jena [42]	Technostress in ICT enabled collaborative learning environment: An empirical study among Indian academician	Information Systems	x	x			
Khedhaouria and Cucchi [38]	Technostress creators, personality traits, and job burnout: A fuzzy-set configurational analysis.	Information Systems	x	x	x		
Kim, Lee, Yun and Im [49]	An examination of work exhaustion in the mobile enterprise environment.	social sciences	x	x	x	x	
Lee [73]	Does stress from cell phone use increase negative emotions at work?	psychology	x				
Ragu-Nathan, Tarafdar, Ragu-Nathan and Tu [5]	The Consequences of Technostress for End Users in Organisations: Conceptual Development and Empirical Validation	Information Systems	x	x	x	x	x
Srivastava, Chandra and Shirish [39]	Technostress creators and job outcomes: theorising the moderating influence of personality traits.	Information Systems	x	x	x	x	x
Stadin, Nordin, Broström, Magnusson Hanson, Westerlund and Fransson [35]	Information and communication technology demands at work: the association with job strain, effort-reward imbalance and self-rated health in different socioeconomic strata	public health	x	x	x	x	x
Stadin, Nordin, Brostrom, Hanson, Westerlund and Fransson [34]	Repeated exposure to high ICT demands at work, and development of suboptimal self-rated health: findings from a 4-year follow-up of the SLOSH study	public health	x	x	x	x	x
Suh and Lee [43]	Understanding teleworkers’ technostress and its influence on job satisfaction	Information Systems	x	x			
Tarafdar, Tu, Ragu-Nathan and Ragu-Nathan [8]	The impact of technostress on role stress and productivity	Information Systems	x	x	x	x	x
Tarafdar, Tu and Ragu-Nathan [40]	Impact of technostress on end-user satisfaction and performance	Information Systems	x	x	x	x	x
Tarafdar, Pullins and Ragu-Nathan [50]	Technostress: negative effect on performance and possible mitigations	Information Systems	x	x	x	x	x
Vayre and Vonthron [41]	Identifying Work-Related Internet’s Uses—at Work and Outside Usual Workplaces and Hours—and Their Relationships With Work–Home Interface, Work Engagement, and Problematic Internet Behaviour	psychology		x			
Wu, Wang, Mei and Liu [44]	Technology-induced job anxiety during non-work time: Examining conditional effect of techno-invasion on job anxiety.	Information Systems		x			

## Appendix B

**Table A2:** Search strategy (PubMed and PubMed Central, slightly adapted for other databases).

(i) (occupational diseases [MH] OR occupational exposure [MH] OR occupational exposure * [TW] OR “occupational health” OR “occupational medicine” OR work-related OR working environment [TW] OR at work [TW] OR work environment [TW] OR occupations [MH] OR work [MH] OR workplace * [TW] OR workload OR occupation * OR worke* OR work place * [TW] OR work site * [TW] OR job * [TW] OR occupational groups [MH] OR employment OR worksite * OR industry)	AND	(ii) (technostress OR techno-stress)]

The * is a conventional function used to search electronic databases.
